# Supplementary material for: Incorporating the Cluster A and V1V2 Targets into a Minimal Structural Unit of the HIV-1 Envelope to Elicit a Cross-Clade Response with Potent Fc-Effector Functions
Source: Vaccines (Basel). 2021 Aug 31;9(9):975. doi: 10.3390/vaccines9090975 (PMC8472903; doi:10.3390/vaccines9090975)
Supplement: Supplementary file 1 [file vaccines-09-00975-s001.zip › Sup. Figure legends.pdf]

Figure S1 (**A**) Full length clade A/E 93TH057 gp120 amino acid sequence with regions used to make ID2-V1V2 colored in blue with the V1V2 loop in green. Residues Val^{65} and Ser^{115} which are mutated to cysteine in ID2-V1V2 to make a stabilizing disulfide bond are highlighted in red. (**B**) The amino acid sequence of clade A/E 93TH057 ID2-V1V2 colored as in (**A**). The Gly-Gly linker that replaces the gp120 outer domain and the ID2-V1V2 stabilizing disulfide bond are colored in red. (**C**) The codon optimized DNA sequence of clade A/E 93TH057 ID2-V1V2.

Figure S2. Sub-cutaneous immunization analysis. Sera from mice immunized subcutaneously with ID2 or ID2-V1V2 was analyzed for ADCC against (**A** gp120 core coated target cells and (**B** Bal gp120 coated target cells. (**C**) CH59 competition ELISA (**D**) sera binding to wild type and Nef-VPU- ADA virally infected cells. **(E**) ADCC against wild type and Nef-VPU- ADA virally infected cells.
