# Supplementary material for: Incorporating the Cluster A and V1V2 Targets into a Minimal Structural Unit of the HIV-1 Envelope to Elicit a Cross-Clade Response with Potent Fc-Effector Functions
Source: Vaccines (Basel). 2021 Aug 31;9(9):975. doi: 10.3390/vaccines9090975 (PMC8472903; doi:10.3390/vaccines9090975)
Supplement: Supplementary file 1 [file vaccines-09-00975-s001.zip › Supplementary Materials and Methods.pdf]

Supplementary Materials and Methods

ADCC

HIV-1 Infected cell ADCC was carried out as previously described [32, 33]. Briefly, primary CD4+ T cells were infected with ADA-Env-based NL4.3 GFP viruses containing intact (WT) or defective *nef* and *vpu* genes (N-U-). Infected cells were stained with viability (AquaVivid; Thermo Fisher Scientific, Waltham, MA, USA) and proliferation (cell proliferation dye eFluor670; Thermo Fisher Scientific, Waltham, MA, USA) markers for use as target cells. CD4 T cells were then mixed with PBMCs at a ratio of 10:1 and pooled mouse sera (1:1000) was added to the wells. Plates were centrifuged briefly at 300× *g* before incubating at 37 °C for 5 - 6 h. Cells were then fixed in 2% PBS-formaldehyde solution and samples were acquired on an LSRII cytometer (BD Biosciences, Franklin Lakes, NJ, USA), data was analysed using FlowJo (Version 10, Tree Star, Ashland, OR, USA).
