# Supplementary figures and images for: Incorporating the Cluster A and V1V2 Targets into a Minimal Structural Unit of the HIV-1 Envelope to Elicit a Cross-Clade Response with Potent Fc-Effector Functions
Source: Vaccines (Basel). 2021 Aug 31;9(9):975. doi: 10.3390/vaccines9090975 (PMC8472903; doi:10.3390/vaccines9090975)

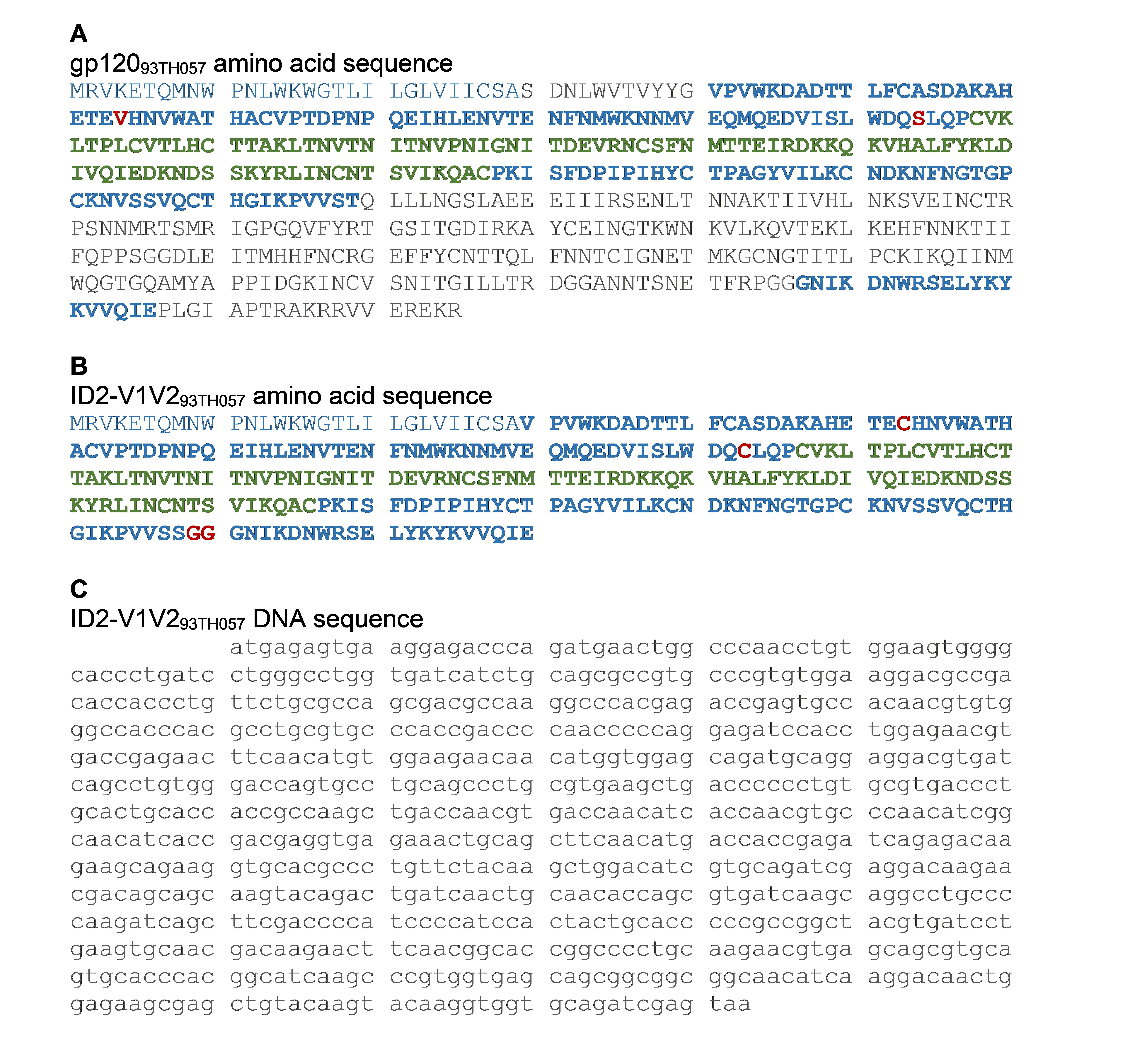

Supplement: Supplementary file 1 [file vaccines-09-00975-s001.zip › Figure S1.JPG]

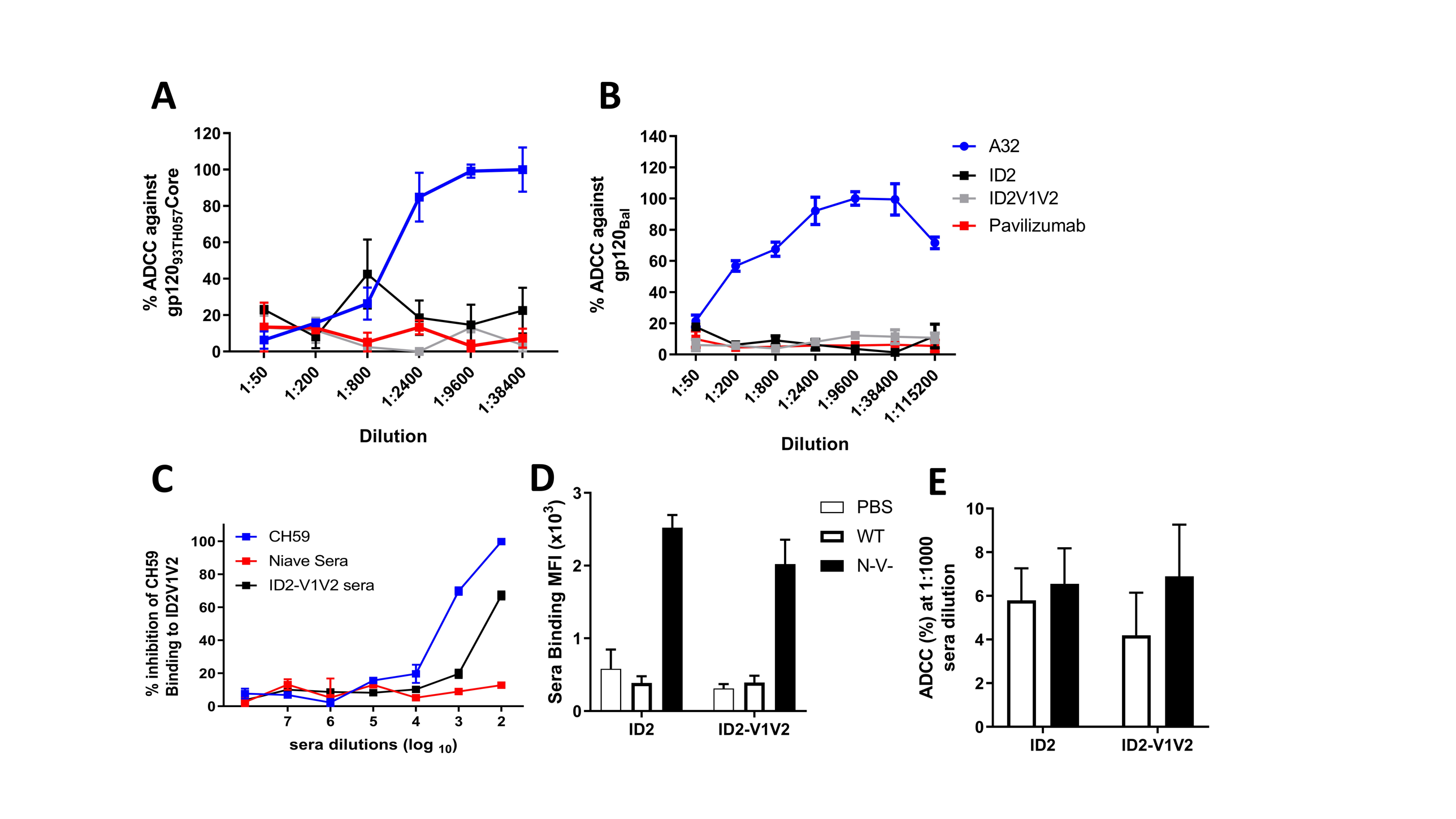

Supplement: Supplementary file 1 [file vaccines-09-00975-s001.zip › Figure S2.JPG]
